# Anomalous Photoelectric Effect of a Polycrystalline Topological Insulator Film

**DOI:** 10.1038/srep05876

**Published:** 2014-07-29

**Authors:** Hongbin Zhang, Jiandong Yao, Jianmei Shao, Hai Li, Shuwei Li, Dinghua Bao, Chengxin Wang, Guowei Yang

**Affiliations:** 1 State Key Laboratory of Optoelectronic Materials and Technologies, Institute of Optoelectronic and Functional Composite Materials, Nanotechnology Research Center, School of Physics & Engineering, Sun Yat-sen University, Guangzhou 510275, Guangdong, P. R. China; 2 These authors contributed equally to this work.

## Abstract

A topological insulator represents a new state of quantum matter that possesses an insulating bulk band gap as well as a spin-momentum-locked Dirac cone on the surface that is protected by time-reversal symmetry. Photon-dressed surface states and light-induced surface photocurrents have been observed in topological insulators. Here, we report experimental observations of an anomalous photoelectric effect in thin films of Bi_2_Te_3_, a polycrystalline topological insulator. Under illumination with non-polarised light, transport measurements reveal that the resistance of the topological surface states suddenly increases when the polycrystalline film is illuminated. The resistance variation is positively dependent on the light intensity but has no relation to the applied electric field; this finding can be attributed to the gap opening of the surface Dirac cone. This observation of an anomalous photoelectric effect in polycrystalline topological insulators offers exciting opportunities for the creation of photodetectors with an unusually broad spectral range. Moreover, polycrystalline topological insulator films provide an attractive material platform for exploring the nature and practical application of topological insulators.

Three-dimensional topological insulators (TIs) represent a novel topological phase of quantum matter that possesses insulating bulk states while also supporting metallic surface states protected by time-reversal symmetry^1^,^2^,^3^. Spin- and angle-resolved photoemission spectroscopy has confirmed that these surface states consist of an odd number of Dirac cones with helical spin-momentum textures^4^,^5^,^6^,^7^. It has been theoretically predicted that these topological surface states should exhibit exotic responses to light such as photon-dressed surface band structures^8^,^9^, spin-polarised electrical currents^10^,^11^,^12^, and photon-induced topological phase transitions^13^,^14^,^15^. Recently, the existence of Floquet-Bloch states on the surfaces of TIs has been demonstrated via time- and angle-resolved photoemission spectroscopy^9^, and polarised-light-induced photocurrents have been observed via transport measurements^10^. However, these studies were performed using single-crystal TIs. Thus far, there have not been any investigations of the interaction between light and polycrystalline TIs. Yet, high-quality polycrystalline TI Bi_2_ (Te, Se)_3_ thin films have been prepared through pulsed-laser deposition (PLD)^16^,^17^,^18^, and several detection experiments have confirmed that polycrystalline TI films provide an attractive material platform for exploring the nature and practical application of TIs^19^,^20^,^21^,^22^.

Here, for the first time, we demonstrate an anomalous photoelectric effect in polycrystalline TI Bi_2_Te_3_ films under illumination with non-polarised light. In stark contrast to the traditional photoelectric effect, in which the resistance decreases under light illumination^23^, we find that the resistance of the surface states suddenly increases when the sample is illuminated. This resistance variation is positively dependent on the light intensity but is independent of the driving voltage. Based on the novel photoresponse features of our prepared polycrystalline TI films, we argue that gap opening of the topological surface states may occur, even under illumination with non-polarised light, resulting in reduced mobility of the surface-state electrons.

## Results

**Figure 1:**
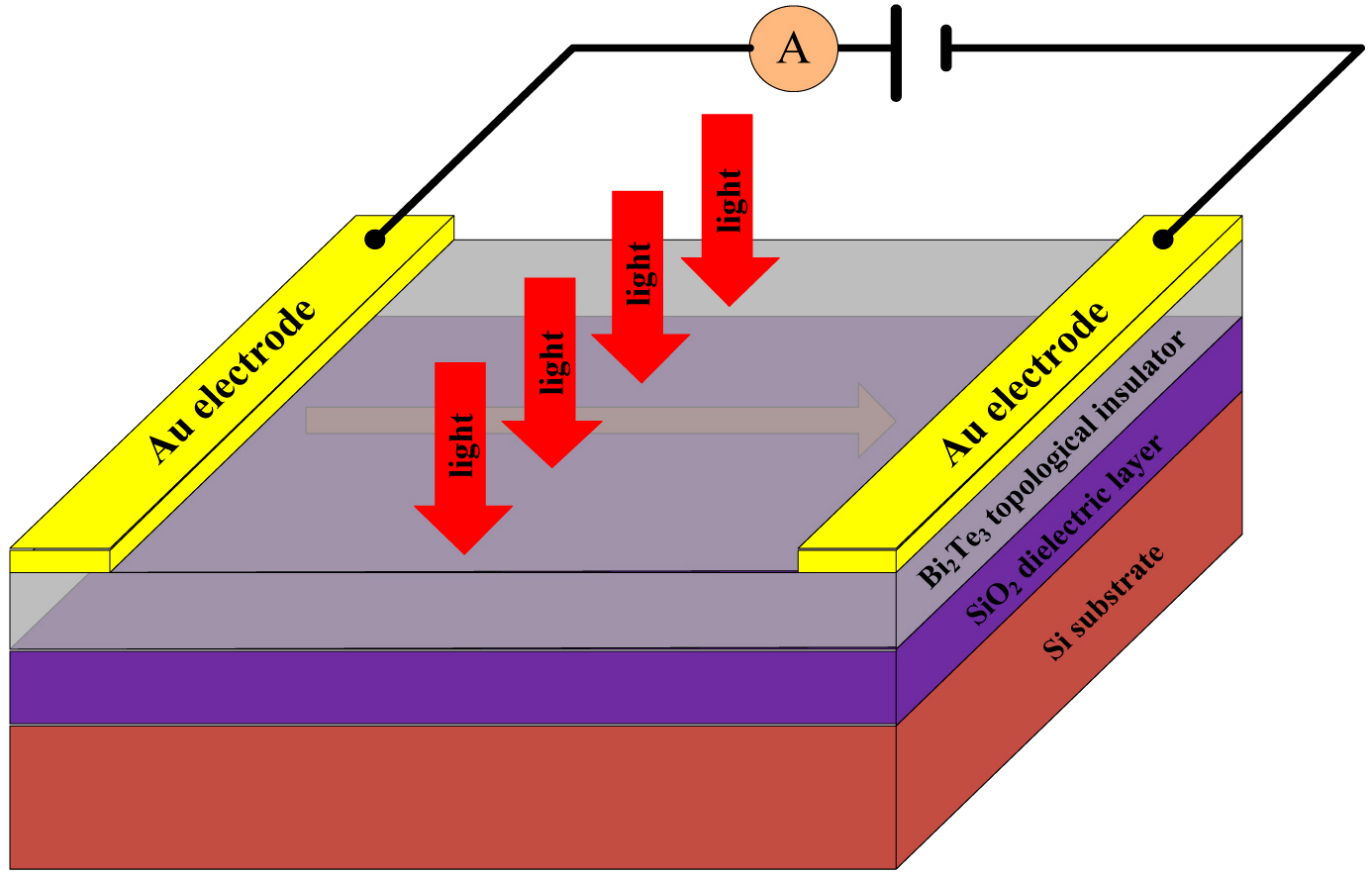
Schematic diagram of the device and the measurement circuitry, consisting of Si substrate, SiO_2_ dielectric layer, Bi_2_Te_3_ polycrystalline topological insulator thin film and Au electrodes. The red arrows represent the incident laser, which illuminates globally and symmetrically on the Bi_2_Te_3_ film.

The transport characteristics of polycrystalline TI Bi_2_Te_3_ films (Fig. S1 in Supplementary Information) were studied using a Keithley 4200-SCS semiconductor characterisation system. The measurements were conducted in sampling mode at room temperature. Lasers with various wavelengths (532, 635 and 1064 nm) were used to illuminate and excite the stacked device. To provide better contact, identical Au electrodes were deposited on the top surface of the as-grown film via ion sputtering using a shadow mask at room temperature. A schematic diagram of the as-prepared device and the measurement circuitry is presented in Figure 1. A characteristic current-voltage (I–V) curve of the device exhibits a perfectly linear dependence, suggesting that an ohmic contact is formed between the Au electrode and the polycrystalline Bi_2_Te_3_ film. Considering that Bi_2_Te_3_ is an excellent thermoelectric material with a large Seebeck coefficient^24^,^25^, we applied symmetrical illumination to avoid interference from the thermoelectric potential caused by a temperature difference between the two sides of the sample during the measurement. To ensure the absence of this effect, before the formal measurements, we illuminated the Bi_2_Te_3_ film without a driving voltage and adjusted the position of the light until the measured voltage between the two electrodes reached zero.

**Figure 2:**
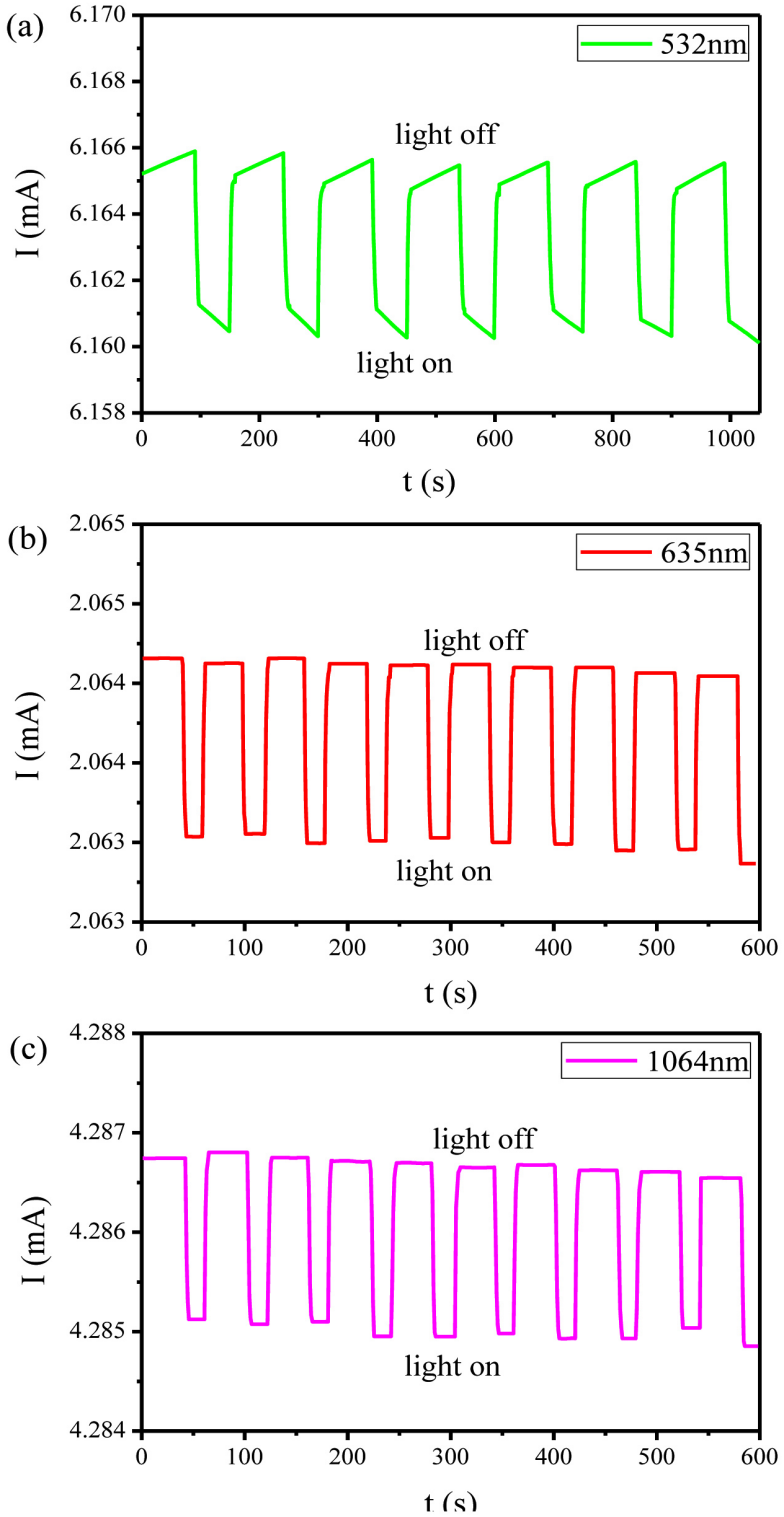
Representative current-time characteristic curves of the anomalous photoelectric effect of Bi_2_Te_3_ topological insulator film under global and symmetrical illumination with lasers wavelength of (a) 532 nm, (b) 635 nm, and (c) 1064 nm, respectively. The power of the incident light is 50 mW and the sport size is 1 cm in diameter. The size of the device is 4 × 4 mm^2^. The regimes with larger current are the light off regimes, while the regimes with lower current are the light on regime, as is indicated in the graph. Obviously, under illumination of excited light, the current jumps downward, indicating that the film changes from low resistance state to high resistance state, contrary to the traditional photoelectric effect.

First, we performed detailed studies of the influence of various types of light on the polycrystalline Bi_2_Te_3_ film. The responses to green (532 nm), red (635 nm) and infrared (1064 nm) light are presented in Figure 2(a), (b) and (c), respectively. As shown in these figures, the Bi_2_Te_3_ film exhibits an intriguing anomalous photoelectric effect. With the traditional photoelectric effect, if the energy of the incident photon is greater than the semiconductor's band gap, the semiconductor will absorb the incident photon and generate electron-hole pairs. Such a change increases the carrier concentration and thus increases the electrical conductivity, i.e., it reduces the resistance of the material. However, compared with the traditional photoelectric effect, our experimental observations indicate a completely opposite response to light illumination. When the light is turned off, the Bi_2_Te_3_ film remains in a state of higher current, henceforth called the low-resistance state (LRS). After global and symmetrical illumination is applied to the film, the current drops sharply within seconds and then remains at a lower value, in a state henceforth called the high-resistance state (HRS). When the light is turned off once more, the current increases rapidly, and the sample returns to the LRS. In summary, when the film is illuminated, its resistance suddenly increases rather than decreases. Because this behaviour is opposite to that observed for the traditional photoelectric effect, we refer to it as an anomalous photoelectric effect (Fig. S2 in Supplementary Information). In fact, the resistance of the film changes much more rapidly than the shift reflected in the experimental data, as the output power of the laser cannot instantaneously change from zero to the target working power or vice versa. Experimentally, these changes require a duration of several seconds, the same magnitude as the shifting time estimated from the curves presented in Figure 2, suggesting that the switching response is much quicker than could be directly measured. Moreover, we further investigated the changes in the current under light illumination over a much longer time scale. The current undergoes a sharp decrease and then remains at a constant value (Fig. S3 in Supplementary Information).

**Figure 3:**
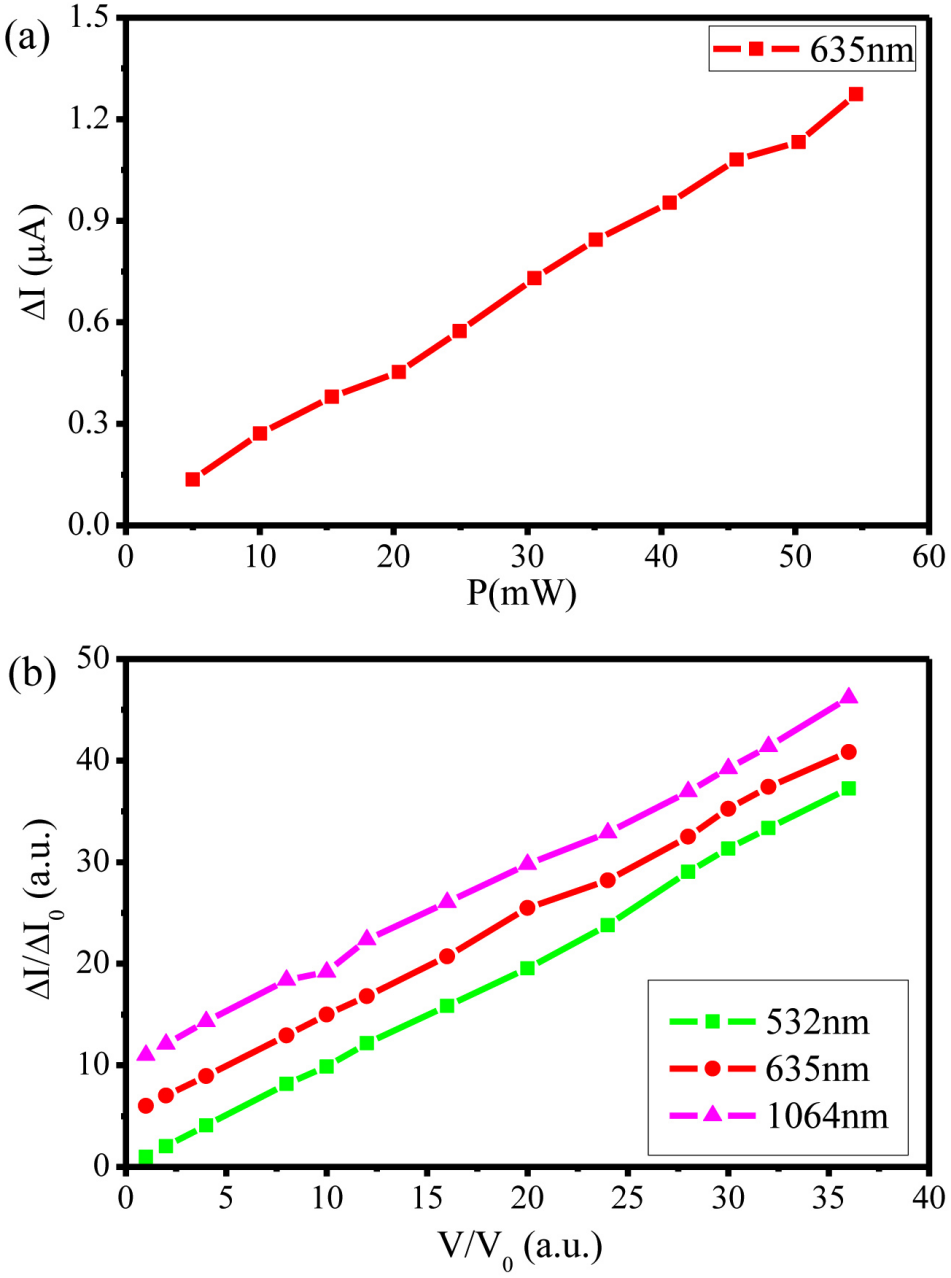
Influence of different parameters on the anomalous photoelectric effect of Bi_2_Te_3_ films. (a) Power-dependent current variation under the illumination of red light and at a driving voltage of 0.3 V. (b) Normalized voltage-dependent current variation for green light, red light, infrared light, respectively. V_0_ (minimum voltage of all data points in the graph) is 0.05 V and I_0_ is the corresponding current. The lines are vertically shifted for clarity.

To gain a deeper understanding of this anomalous photoelectric effect, we further explored the influences of other parameters, including the intensity of the incident light and the driving voltage. First, a series of transport measurements was performed under illumination with red light of various intensities for a constant driving voltage of 0.3 V. As shown in Figure 3(a), the variation in the current seems to be proportional to the light intensity. Namely, the resistance of the film increases with increasing light intensity, suggesting that the opening of gaps is positively correlated with the light intensity. We then performed measurements for a series of driving voltages under illumination with green, red and infrared light. Figure 3(b) presents the normalised voltage-dependent variation in the current for different types of illumination. Nearly identical linear dependencies are observed for the different types of light, indicating that the resistance has no dependence on the driving voltage.

## Discussion

According to our previous transport studies of polycrystalline Bi_2_Te_3_ films, the surface-state electrons always exhibit stable, weak anti-localisation (WAL) even in the presence of strong disorder, while the Hall resistance can exhibit a significantly nonlinear magnetic-field dependence. Moreover, in contrast with the WAL correction in conventional spin-orbit coupling materials, which always become insulating when the disorder exceeds a critical level, the observed WAL quantum transport phenomenon in our Bi_2_Te_3_ polycrystalline films is highly stable. Therefore, this topological delocalisation may provide a convenient basis for determining when our prepared Bi_2_Te_3_ polycrystalline film is in the TI state^21^. Because a TI typically possesses a relatively insulating bulk transport channel and a spin-polarised surface transport channel, the standard TI theory and experimental verifications thereof indicate that the thickness of the topological surface channel is on the order of a few nanometres. Thus, for the in-plane measurement configuration used in this study, the surface-state electrons and bulk-state electrons contribute in parallel. Because of the relatively large bulk-state resistance, the surface-state carriers actually dominate the overall transport in our films, whereas the electrical properties of the bulk are virtually masked by the surface channels. Thus, we attribute the increase in the resistance of our polycrystalline Bi_2_Te_3_ films under light illumination to the opening of gaps in the surface Dirac cone; that is, once the light is switched on during the measurement, an energy gap opens in the surface Dirac cone, leading to a reduction in the surface carrier mobility and therefore contributing to the increased resistance and inducing the anomalous photoelectric effect.

Theoretical calculations have demonstrated that Dirac cones of graphene and TI should open under light illumination^8^,^26^,^27^,^28^. Moreover, such gaps have recently been observed via time- and angle-resolved photoemission spectroscopy of the surface of a Bi_2_Se_3_ thin film^9^. In contrast to the assumption applied in the above theoretical calculations, the light sources used in our experiment are non-polarised rather than being circularly polarised or linearly polarised, which lends additional interest to the gap-opening phenomenon observed herein compared with previous experimental observations and theoretical calculations. It should be particularly noted that the samples used in our transport measurements are polycrystalline films instead of single crystals. Polycrystalline Bi_2_Te_3_ films exhibit the same rhombohedral crystal structure in the space group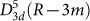 as single crystals, and the layered crystal structure of polycrystalline Bi_2_Te_3_ films also satisfies space-inversion symmetry. Thus, our polycrystalline Bi_2_Te_3_ films should possess a similar Dirac-cone structure on their surfaces, and the formation mechanism of topological surface states should be nearly identical to that for single crystals. Furthermore, according to the traditional theory, electron scattering at the grain boundaries in the polycrystalline films should also comply with time-reversal symmetry, and thus, the scattering of surface electrons in the polycrystalline films should maintain destructive interference at all times, resulting in the suppression of backscattering and preservation of the topological protection effect. Therefore, based on the fact that they exhibit the same crystal structure and energy-band structure as well as similar electron-transport properties, polycrystalline Bi_2_Te_3_ films should display a light response similar to that of single crystals, and thus, a light-induced gap opening in the Dirac cone is expected in our prepared polycrystalline Bi_2_Te_3_ films.

Due to intrinsic defects, the Fermi energy of the undoped Bi_2_Te_3_ films may lie within a bulk conduction band, making the system essentially metallic; thus, the thermal effect induced by illumination can produce a positive resistance via enhanced electron-photon scattering. However, an instantaneous positive resistance effect cannot be observed due to the long relaxation times, especially during the recovery process after the illumination is removed, because an extremely long-lived tail is common in materials that demonstrate a heat-induced negative photocurrent. Moreover, there is no saturated, steady photocurrent state that arises with increasing illumination time^29^,^30^. By contrast, the photoresponse of our prepared Bi_2_Te_3_ film is quite different: after an initial rapid decline, a steady-state photocurrent is quickly attained, and a nearly symmetrical relaxation to the dark conduction state follows once the illumination is removed. Thus, the transient photoresponse process is rapid in nature. Under continuous illumination, the current in our films drops markedly and can remain at a low, saturated level. Therefore, although a small thermal contribution cannot be separated or completely ruled out in our study, the lack of a long tail in the transient response nevertheless reveals that the negative photocurrent signal is not a predominantly thermal artefact. Moreover, using high-resolution angle-resolved photoemission spectroscopy, Pan *et al*.^31^ recently observed an extremely weak broadening of the topological surface state with increasing temperature, with no anomalies in the dispersion of the state. These results imply an exceptionally weak electron-phonon interaction on the surface of Bi_2_Te_3_ films, providing further convincing evidence for the exclusion of a thermal origin for the resistance observed in our experiment.

According to previous studies, negative persistent photoconductivity can most likely be attributed to the trapping of electrons by an empty localised state of random fluctuations in the local potential in the barrier. Thus, in conventional non-degenerate semiconductors, it is necessary to introduce at least two gap states of donor and acceptor types, filled or partially filled, to explain observations of a negative photoconductivity effect^32^,^33^,^34^. When the light is turned on, electrons are excited from the valence band to an upper impurity level, which is not completely filled with electrons, thus leaving a hole in the valence band. The hole rapidly recombines at a lower impurity level, which is normally filled with electrons, and an electron from the conduction band then becomes trapped at the lower level. Thus, the de-excitation of electrons from the conduction band limits the build-up of carrier concentration, reducing the conductivity below the dark level and resulting in an anomalous negative photoresponse. Meanwhile, at increased photon energies, an additional positive photocurrent is expected because of the direct excitation of carriers from the lower level to the conduction band. However, in our photoelectric measurements, only a positive resistance effect is observed with increasing illumination pulse intensity; thus, the model described above is not consistent with our experimental data.

In addition, the negative photosignal described by the above model is usually strongly dependent on temperature because the number of conduction electrons available to be trapped drastically decreases as the temperature rises, and the increased rate of thermal ionisation of electrons from the upper level to the conduction band may cause the negative photosignal to essentially vanish at certain high temperatures; however, the anomalous photoelectric effect observed in our experiments is particularly pronounced at room temperature. Furthermore, according to the gap-state transition model, the photoresponse should be dependent on wavelength, and transient negative photoconduction should be observed at a specific photon energy. We observed, however, that the intrinsic photoconductivity response for our prepared Bi_2_Te_3_ films persisted over a broad spectral range. Thus, we note that the previously proposed model, which attributes the negative photoeffect to the capture of photoexcited mobile electrons by trapping levels, fails to provide a satisfactory interpretation of our observations in multiple respects.

**Figure 4:**
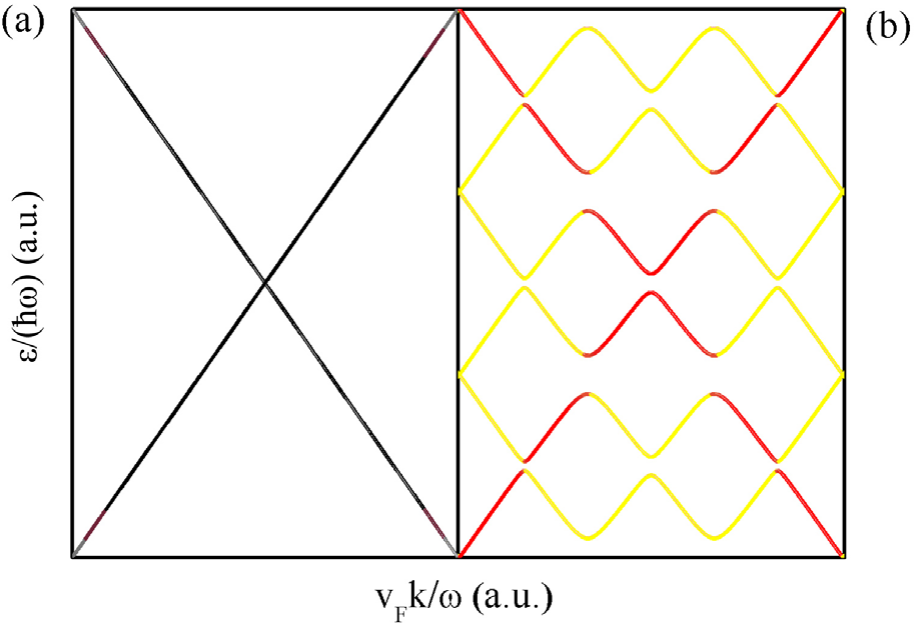
Dispersion relations of the surface state of a topological insulator. (a) Perfect Dirac cone before light irradiation, (b) quasienergy spectrum with gaps at specific momentum under circularly polarized light irradiation.

Based on our experimental data, we would like to emphasise that the observed anomalous photoeffect is an inherent feature of Bi_2_Te_3_ TI films under illumination. We suggest a new mechanism, arising from the gap opening of the Dirac cone, that is capable of producing a sharp decrease in surface electron mobility, thereby contributing to the total positive resistance effect compared to the dark state. As shown in Figure 4(a), the surface state of a topological insulator shows a perfect Dirac cone. Because of the spin-momentum-locked Dirac-cone structure, the surface-state electrons of a TI film usually acquire a π Berry phase, which can suppress backscattering processes through destructive interference between two closed paths with time-reversal symmetry, thereby providing higher surface electron mobility. However, at the onset of illumination, the photoexcitation of the surface electrons may induce a gap at the Dirac point (Figure 4(b)), resulting in photon-assisted backscattering due to the weakened topological protection, which may lead to a rapid decrease in surface carrier mobility^34^,^35^.

The conductivity of a film can be expressed as *σ* = *enu*, where e is the electronic charge, n is the carrier density, and *μ* is the electron mobility. Thus, a reduction in surface electron mobility in the excited states caused by enhanced backscattering, such as the scattering attributable to interface roughness under illuminated conditions, may ultimately produce an overall negative photoconductance. Furthermore, the electron mobility is important in determining the operating speed of materials and devices; thus, we surmise that the rapid response of the photoelectric effect observed in our films must reflect the decreased mobility in the excited states accompanied by the gap opening of the Dirac cone. Moreover, the periodic changes in the current observed under cyclical illumination demonstrate that this phenomenon is highly repeatable, and only changes in mobility induced by the opening of the band gap could result in such cyclic and repeatable behaviour over several on-off cycles.

According to the Floquet theory, the gap opened at the Dirac point is described by 
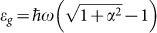
27, where 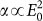; *E*_0_ is the amplitude of the electric-field strength related to the power density of the light, 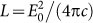; and *c* is the speed of light. If the power of the light is not strong, as in our experiment, then 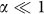 and 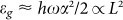. Therefore, the energy gap that is opened at the Dirac point under light illumination is proportional to the power density of the light. Thus, the variation in the current should exhibit a positive dependence on the light intensity. The results of our experiment are qualitatively consistent with this theoretical calculation. This agreement between theory and experiment confirms that the reduction in current under light illumination in our polycrystalline TI thin films originates from the photon-induced gap at the Dirac point.

In the case of our transport experiments, only laser light of various wavelengths was used; no polarised light was employed. Thus, further experiments will be necessary to confirm whether the observed anomalous photoelectric effect becomes stronger or remains unchanged in the case of polarised illumination. In the near future, we will conduct more detailed studies of the interactions between our polycrystalline TI films and various types of polarised light, including linearly and circularly polarised light. In the existing literature, only the photoelectric features of topological surface electrons under polarised light have been reported; there have not been any reports related to the photoresponse under non-polarised light. Therefore, we propose our own interpretations of our results. On the basis of previous observations and the analyses of related experimental phenomena, we believe that the positive resistance effect observed in our polycrystalline Bi_2_Te_3_ TI films originates from the photon-induced gap at the Dirac point.

In summary, these findings related to an anomalous photoelectric effect in Bi_2_Te_3_ polycrystalline TI films suggest that polycrystalline TI films may be appropriate for achieving photodetectors with an unusually broad spectral range while also providing an attractive material platform for exploring the nature of TIs.

## Methods

We used pulsed-laser deposition (PLD) to prepare polycrystalline TI Bi_2_Te_3_ thin films^16^,^19^. In our experiments, the deposition parameters were as follows. The base pressure of the growth chamber was below 1 × 10^−4^ Pa. The substrates used in the PLD growth were (100)-oriented single-crystal Si wafers with a 300-nm-thick SiO_2_ dielectric layer isolating the Bi_2_Te_3_ film from the Si substrate to avoid interference from the substrate in the transport measurements. The target material consisted of highly pure and uniform Bi (99.999%) and Te (99.999%) elements with a Bi:Te atomic ratio of 2:3. Prior to loading into the growth chamber, the substrates were first cleaned in acetone via ultrasonication for 15 minutes for the removal of organic contaminants. Pre-growth annealing at 500°C for 40 minutes was performed to remove native oxides from the substrate. High-quality polycrystalline thin films were deposited at an optimised substrate temperature of 300°C for 20 minutes, and the working pressure was set to 40 Pa, with Ar_2_ flowing at a rate of 50 sccm as the working gas.

## Supplementary Material

Supplementary Information Anomalous Photoelectric Effect of a Polycrystalline Topological Insulator Film
