# Supplementary material for: Anomalous Photoelectric Effect of a Polycrystalline Topological Insulator Film
Source: Sci Rep. 2014 Jul 29;4:5876. doi: 10.1038/srep05876 (PMC5376173; doi:10.1038/srep05876)
Supplement: Supplementary Information — Anomalous Photoelectric Effect of a Polycrystalline Topological Insulator Film [file srep05876-s1.doc]

**Supplementary Information**

**Anomalous Photoelectric Effect of a Polycrystalline Topological Insulator Film**

Hongbin Zhang, Jiandong Yao, Jianmei Shao, Hai Li, Shuwei Li, Dinghua Bao, Chengxin Wang & Guowei Yang

State Key Laboratory of Optoelectronic Materials and Technologies, Institute of Optoelectronic and Functional Composite Materials, Nanotechnology Research Center, School of Physics & Engineering, Sun Yat-sen University, Guangzhou 510275, Guangdong, P. R. China

**Materials and methods**

We use pulsed-laser deposition (PLD) to prepare polycrystalline TI Bi_2_Te_3_ thin films (*1-2*). In our experiments, the deposition parameters are as follows. The base pressure of the growth chamber is better than 1×10^-4^ Pa. The substrates used in the PLD-growth are (100) oriented single crystal Si wafers with a 300 nm-thick SiO_2_ dielectric layer which isolates the Bi_2_Te_3_ film and Si substrate and avoids the interference of the substrate on the transport measurements. The target material consists of highly pure and uniform Bi (99.999%) and Te (99.999%) elements with the Bi: Te atomic ratio of 2:3. Prior to loading into the growth chamber, the substrates are firstly cleaned in acetone by ultrasonic wave for 15 minutes to remove the organic contaminations. Pre-growth annealing at 500ºC for 40 minutes is performed to remove the native oxides from the substrate. High-quality polycrystalline thin films are deposited at the optimized substrate temperature of 300ºC for 20 minutes, and the working pressure is set at 40 Pa with flowing Ar_2_ as the working gas in the rate of 50 sccm.

**Characterizations of the PLD-growth Bi_2_Te_3_ film**

Scanning electron microscope (SEM), Raman spectroscopy and X-ray diffraction (XRD) were conducted to study the morphology and structure of the PLD-growth Bi_2_Te_3_ film, as shown in Fig. S1. Fig. S1(a) presents a typical SEM image. It seems that the film possesses smooth and continuous surface morphology in large scale. Besides, grains are evenly distributed in the film, indicating a high degree of homogeneity of the polycrystalline thin films. Fig. S1(b) shows a representative Raman spectroscopy with the 514 nm excitation laser. There are two characteristic peaks at 102.2 cm^-1^ and 135.1cm^-1^ , which are consistent with the vibration mode $E_{g}^{2}$ and $A_{1g}^{2}$, respectively. Similar results have been observed by other groups (*3-6*). It is worthy to note that the $A_{1g}^{1}$ vibration mode (62.5 cm^-1^) is out of our measurement range. X-ray diffraction pattern is presented in Fig. S1(c). It only shows the (003) family diffraction peaks, indicating that the sample possesses the rhombohedral structure and is highly c-axis oriented, consistent with the results of other groups (*6-8*). All of the above-mentioned characterizations indicate that the PLD-growth Bi_2_Te_3_ film possesses high crystal quality and provides an attractive material platform to investigate the properties of topological insulator.

**Exclusion of photo-induced thermal-resistance effect**

In our case, one possible reason for the resistance’s increase is the photon-induced thermal-resistance effect. It is originated from the temperature increase due to the radiation of incident light, which exacerbates phonon vibration and thus results in an increase in the electron-photon scattering rate. Ultimately, it increases the resistance of the sample. To investigate whether our experimental results are originated from the thermal-resistance effect, we need to understand the principle of it.

As we all know, metal and semiconductor materials exhibit completely different thermal-resistance effect. Generally, the temperature-dependent metallic thermal-resistance is given by

$R_{T}=R_{T_{0}}[1+\alpha(T-T_{0})]$,

while the temperature-dependent semiconducting thermal-resistance is given by

$R_{T}=Ae^{\frac{B}{T}}$.

*R_T_* is the resistance at *T*, *R_0_* is the resistance at *T_0_*, *α* is the resistance temperature coefficient (*α* can be treated as a positive constant at a small temperature range), A and *B* are constants depending on the structure and material of the semiconductor. Therefore, the resistance of metal is positively while that of semiconductor is negatively dependent on the temperature, as shown in Fig. S2.

When a sample is exposed to light illumination, its temperature will rise gradually over time owing to the light radiation. If the resistance’s increase in our Bi_2_Te_3_ films is originated from the photon-induced thermal-resistance effect, the resistance will gradually increase and the current should decrease gradually over time. However, as shown in Fig. S3, in our study, the current-time characteristic curves perform totally differently from that expected for the metallic or semiconductor thermal-resistance effect. In our case, when the light is turned on, the current suddenly decreases. Then, it almost remains a constant value even over a time as long as 500 s. Therefore, the rise in resistance could not be caused by the increase in temperature. Thus, we conclude that the resistance’s increase is originated from the opening of gaps in the surface Dirac cone, and the photon-induced thermal-resistance is negligible compared to it.

**Figure S1**. Characterizations of the PLD-growth polycrystalline topological insulator Bi_2_Te_3_ film. (a) Low-magnification SEM image, scale bar: 1um. (b) Raman spectrum with the 514nm excitation laser. The two characteristic peaks are the $E_{g}^{2}$ (102.2cm^-1^) and $A_{1g}^{2}$ (135.1cm^-1^) mode respectively. The side views of two vibration modes are shown in the inset. (c) 2θ-ω X-ray diffraction pattern. It only shows (003) family diffraction peaks of Bi_2_Te_3_. All the characterizations suggest that the PLD-growth Bi_2_Te_3_ film is of high quality.


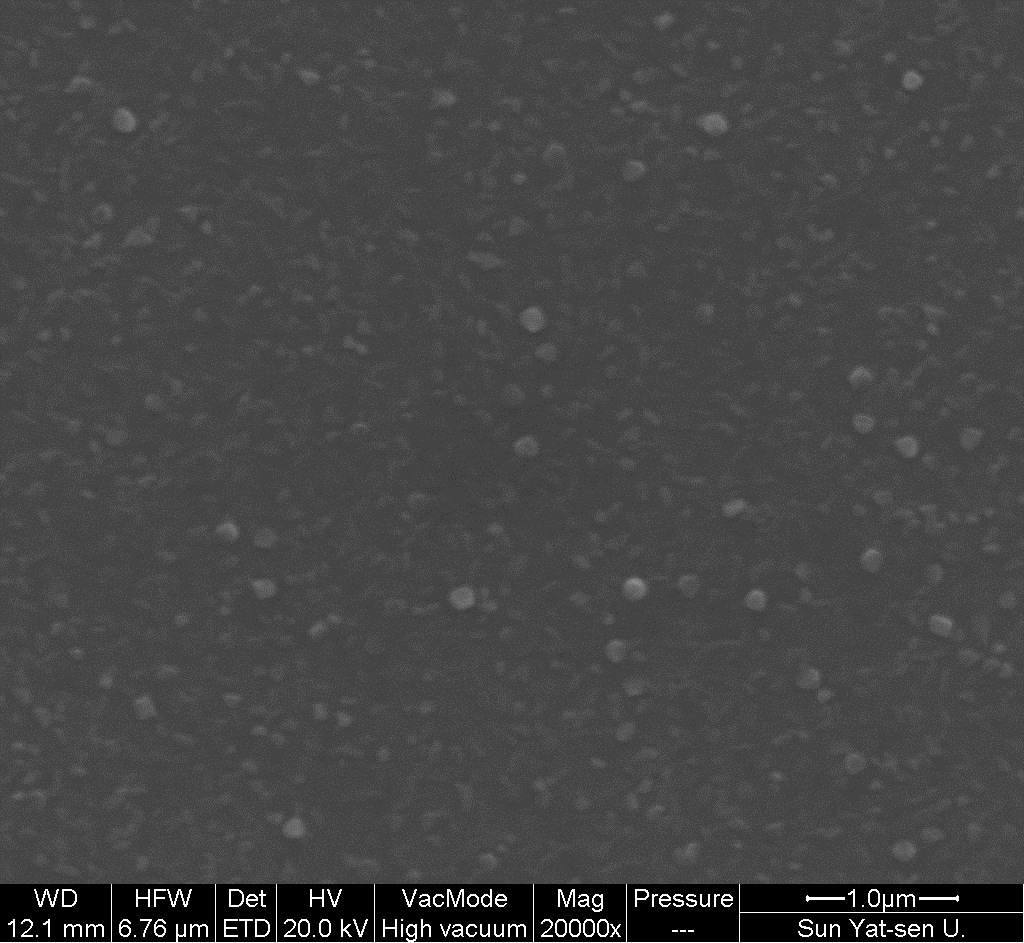


(a)

(b)

**Figure S2**. Representative resistance-temperature characteristic curves of metal and semiconductor materials.

**Figure S3**. Representative current-time characteristic curves of the anomalous photoelectric effect under global and symmetrical illumination of large time scale with lasers wavelength of (a) 532 nm, (b) 635 nm, and (c) 1064 nm，respectively.

**References**

[1] H. B. Zhang, H. L. Yu, G. W. Yang, *Europhys. Lett*. **95**, 56002 (2011).

[2] H. B. Zhang *et al. Adv. Mater.***24**, 132-136 (2012).

[3] W. Richter, H. Kohler, C. R. Becker, *Phys. Stat. Sol.* (b) **84**, 619-628 (1977).

[4] D. Teweldebrhan, V. Goyal and A. A. Balandin, *Nano. Lett.* **10**,1209-1218 (2010).

[5] R. He *et al.* *Nanotechnology.* **23**, 455703 (2012).

[6] X. Liu *et al.* *Appl. Phys. Lett.* **99**, 171903 (2011).

[7] A. Bailini *et al.* *Applied Surface Science*. **254**, 1249-1254 (2007).

[8] S. E. Harrison *et al.* *Appl. Phys. Lett.* **102**, 171906 (2013).
